# Discovery of *GJC1* (Cx45) as a New Gene Underlying Congenital Heart Disease and Arrhythmias

**DOI:** 10.3390/biology12030346

**Published:** 2023-02-21

**Authors:** Yan-Jie Li, Juan Wang, Willy G. Ye, Xing-Yuan Liu, Li Li, Xing-Biao Qiu, Honghong Chen, Ying-Jia Xu, Yi-Qing Yang, Donglin Bai, Ri-Tai Huang

**Affiliations:** 1 Department of Cardiology, Shanghai Chest Hospital, School of Medicine, Shanghai Jiao Tong University, Shanghai 200030, China; 2 Department of Cardiology, Xuhui District Central Hospital, Zhongshan-Xuhui Hospital, Fudan University, Shanghai 200030, China; 3 Department of Cardiology, Longhua Hospital, Shanghai University of Traditional Chinese Medicine, Shanghai 200032, China; 4 Department of Physiology and Pharmacology, Schulich School of Medicine & Dentistry, The University of Western Ontario, London, ON N6A 5C1, Canada; 5 Department of Pediatrics, Tongji Hospital, School of Medicine, Tongji University, Shanghai 200065, China; 6 Key Laboratory of Arrhythmias, Ministry of Education of China, School of Medicine, Tongji University, Shanghai 200092, China; 7 Department of Cardiology, Shanghai Fifth People’s Hospital, Fudan University, Shanghai 200240, China; 8 Cardiovascular Research Laboratory, Shanghai Fifth People’s Hospital, Fudan University, Shanghai 200240, China; 9 Central Laboratory, Shanghai Fifth People’s Hospital, Fudan University, Shanghai 200240, China; 10 Department of Cardiovascular Surgery, Renji Hospital, School of Medicine, Shanghai Jiao Tong University, Shanghai 200127, China

**Keywords:** congenital heart disease, cardiac septal defect, arrhythmia, atrioventricular block, molecular genetics, gap junction channel, *GJC1*, patch clamp electrophysiology

## Abstract

**Simple Summary:**

Congenital heart disease is associated with substantial mortality and morbidity as well as socioeconomic burden, and increasing research underscores the genetic basis for it. In this study, a genome-wide genotyping with microsatellite markers followed by linkage analysis was performed in a Chinese family suffering congenital heart disease and arrhythmias, and a novel genetic locus linked to the disease was located on chromosome 17q21.31-q21.33. Sequencing assays of the candidate genes at the locus revealed a novel heterozygous mutation in the *GJC1* gene coding for Cx45, NM_005497.4:c.550A>G;p.R184G, which was in co-segregation with the disease in the whole family and was not detected in 516 control individuals. Electrophysiological analyses showed that the mutation significantly diminished the coupling conductance in homomeric cell pairs (R184G/R184G) and in cell pairs expressing R184G/Cx45 or R184G/Cx43, in a dominant-negative mode. Fluorescent dye uptake experiments demonstrated no apparent change of the R184G mutation on the Cx45 hemichannels. These findings define a new genetic locus for congenital heart disease and arrhythmias on chromosome 17q21.31-q21.33 and indicate *GJC1* as a new gene predisposing to the disease.

**Abstract:**

As the most prevalent type of birth malformation, congenital heart disease (CHD) gives rise to substantial mortality and morbidity as well as a socioeconomic burden. Although aggregating investigations highlight the genetic basis for CHD, the genetic determinants underpinning CHD remain largely obscure. In this research, a Chinese family suffering from autosomal dominant CHD (atrial septal defect) and arrhythmias was enrolled. A genome-wide genotyping with microsatellite markers followed by linkage assay as well as sequencing analysis was conducted. The functional effects of the discovered genetic mutation were characterized by dual patch-clamp electrophysiological recordings in N2A cells and propidium iodide uptake assays in HeLa cells. As a result, a novel genetic locus for CHD and arrhythmias was located on chromosome 17q21.31-q21.33, a 4.82-cM (5.12 Mb) region between two markers of D17S1861 and D17S1795. Sequencing assays of the genes at the mapped locus unveiled a novel heterozygous mutation in the *GJC1* gene coding for connexin 45 (Cx45), NM_005497.4:c.550A>G;p.R184G, which was in co-segregation with the disease in the whole family and was not observed in 516 unrelated healthy individuals or gnomAD. Electrophysiological analyses revealed that the mutation significantly diminished the coupling conductance in homomeric cell pairs (R184G/R184G) and in cell pairs expressing either R184G/Cx45 or R184G/Cx43. Propidium iodide uptake experiments demonstrated that the Cx45 R184G mutation did not increase the Cx45 hemichannel function. This investigation locates a new genetic locus linked to CHD and arrhythmias on chromosome 17q21.31-q21.33 and indicates *GJC1* as a novel gene predisposing to CHD and arrhythmias, implying clinical implications for prognostic risk assessment and personalized management of patients affected with CHD and arrhythmias.

## 1. Introduction

Congenital heart disease (CHD), which derives from aberrant morphogenesis of the heart and endo-thoracic great blood vessels during embryonic development, represents the most frequent type of birth malformation globally in humans, occurring in about 1% of live births, and accounts for approximately one-third of all birth deformities [1]. The prevalence of CHD in newborns rises to approximately 3% when the congenital bicuspid aortic valve is included [2]. Based on specific anatomic deformities, CHD has been clinically classified into over 25 distinctive entities, including tetralogy of Fallot (TOF), Ebstein’s anomaly, atrial septal defect (ASD), truncus arteriosus, ventricular septal defect (VSD), coarctation of the aorta, interrupted aortic arch, double outlet right ventricle (DORV), endocardial cushion defect (ECD), atrioventricular septal defect (AVSD), hypoplasia of the left ventricle, aortic stenosis, transposition of the great arteries, anomalous pulmonary venous connection, and patent ductus arteriosus [2]. Although slight lesions may resolve spontaneously [2], severe CHD, which accounts for almost one-third of all types of CHD [1], requires timely medical intervention or surgery, and otherwise may lead to diminished exercise performance and degraded health-associated quality of life [3,4,5,6,7], delayed central nervous development and brain damage [8,9,10], ischemic cerebral stroke [11,12], pulmonary arterial hypertension and impaired pulmonary function [13,14,15,16], chronic kidney disease and acute renal injury [17,18,19,20], infective endocarditis [21,22,23,24], aortic dissection and rupture [25], chronic heart failure [26,27], cardiac dysrhythmias [28,29,30], and cardiac premature demise [31,32,33,34]. Over the past several decades, significant improvement in drug therapy and surgical treatment of CHD has been achieved, which remarkably changes the natural history of severe CHD, allowing over 90% of CHD newborns to survive into adulthood [1]. This brings about an ever-growing population of adult CHD patients reaching fertile age, and presently CHD adults outnumber CHD children, constituting 60% of the total population with CHD [1,35]. Nevertheless, the death rate of adults with repaired CHD is much higher in comparison with the general population, and CHD-related cardiac and extra-cardiac comorbidities such as cardiac arrhythmia, heart failure, and neurodevelopmental disability remain substantially increased, even after effective treatment [1,35,36,37]. As such, CHD confers an ever-increasing burden of cardiovascular disease [1]. Despite its clinical importance, the causes of CHD are largely undefined.

The heart is well known as the first functioning organ formed during embryonic genesis in vertebrates, and cardiac morphogenesis undergoes sophisticated biological processes, including expansion, differentiation, apoptosis, and remodeling [38,39]. Such processes are precisely regulated by cellular genes expressed at distinct stages of embryogenic cardiogenesis [39], and both non-heritable environmental precipitating factors and inherited pathogenic components may interfere with the cardiac developmental process, resulting in CHD [40,41,42,43,44,45,46]. The already-established non-heritable risk factors responsible for CHD include maternal obesity, diabetes mellitus, viral infection, folate deficiency, autoimmune disorder, and exposure to toxic chemicals as well as ionizing radiation during pregnancy [1,2,40]. However, environmental causes are identifiable only in 2% of CHD patients [47], and aggregating epidemiological evidence convincingly suggests that heritable causes play a critical role in the majority of CHD [44,45,46]. Genetic defects responsible for CHD include aneuploidy (presence of an additional chromosome, absence of a chromosome, or deletion/duplication of one arm of a chromosome), copy number variation (deletion or duplication of a segment of a chromosome), and genetic mutations [44,45,46]. To date, pathogenic mutations in over 100 genes have been involved in the occurrence of CHD, of which most encode cardiac transcription factors, cell adhesion molecules, signaling pathway proteins, and cardiac structural proteins essential for cardiovascular morphogenesis [44,45,46,48,49,50,51,52,53,54,55,56,57,58,59,60,61,62,63,64,65,66,67,68,69]. However, CHD is of pronounced genetic heterogeneity, and the genetic determinants causative for CHD in a significant proportion of cases remain to be identified.

In this study, to identify a new gene likely responsible for CHD, a four-generation pedigree affected by autosomal dominant CHD as well as arrhythmias was enlisted from the Chinese Han ethnicity population. After a genome-wide genotyping with microsatellite markers followed by linkage assay, a new genetic locus for CHD and arrhythmias was located on chromosome 17q21.31–q21.33. Sequencing assays of the genes at the mapped locus in the family and functional investigation in vitro led to the identification of *GJC1* as a novel gene likely to play a role in CHD and arrhythmias. Obviously, the current study has provided new insight into the molecular mechanisms of the disease.

## 2. Materials and Methods

### 2.1. Recruitment of Research Subjects and Extraction of Genomic DNA

In the present research, a 40-member family spanning four generations affected by CHD and atrioventricular block (AVB) was recruited from the Han ethnicity population in China (designated arbitrarily as Family 1). A total of 516 unrelated ethnically matched individuals, who were healthy with a negative family history of CHD, were recruited as controls (226 females and 290 males, with a mean age of 15 years, varying from 1 year to 60 years of age). All study participants underwent in-depth clinical assessment by cardiologists, including a complete review of personal history as well as family history, thorough physical examination, echocardiography with Doppler color flow imaging (transthoracic and/or transesophageal), electrocardiography, and routine laboratory tests. For the family members who died, their medical records were reviewed. Clinical diagnosis and pathologic categorization of CHD were implemented according to the echocardiographic images and cardiac interventional or surgical findings. This study was performed in conformity with the ethical tenets formulated in the Declaration of Helsinki. Study protocols were reviewed and approved by the ethical committee of Tongji Hospital, affiliated to Tongji University, Shanghai. Informed consent was signed by the adult subjects and the legal guardians of the adolescents prior to enrollment of the research subjects. A peripheral venous blood specimen (6.0 mL) was prepared from each of the research participants, and genomic DNA was purified from venous blood leukocytes utilizing the Gentra Puregene Blood Kit (Qiagene, Hilden, Germany).

### 2.2. Genome-Wide Genotyping with Genetic Markers and Linkage Assay

A genome-wide scan to genotype the pedigree members from Family 1 with CHD and arrhythmias was conducted with a linkage mapping kit (Applied Biosystems, Foster City, CA, USA), utilizing 392 fluorescently labeled microsatellite markers, which were distributed over the 22 autosomes and the X chromosome, spacing at a mean resolution of roughly 10 cM intervals. Additional microsatellite markers near a positive chromosomal region were selected to finely map the genetic locus linked to the disease. Amplification of markers by polymerase chain reaction (PCR) was performed with a DNA polymerase kit (Applied Biosystems, Foster City, CA, USA) under a thermal cycler (Applied Biosystems, Foster City, CA, USA). The fluorescence-labeled amplified products were electrophoresed on a genetic analyzer (Applied Biosystems, Foster City, CA, USA) as per the manufacturer’s manual, and analyzed with the Genescan software (Applied Biosystems, Foster City, CA, USA). Alleles were analyzed with the Genotyper software (Applied Biosystems, Foster City, CA, USA). Genetic linkage analysis and calculation of the two-point logarithm of odds (LOD) score between each marker and the disease locus were completed as described elsewhere [70,71,72]. Haplotypes of Family 1 suffering from CHD were constructed, employing the Cyrillic software (Cherwell Scientific, Oxford, UK) to suggest the shared chromosomal fragments among the affected family members, hereby confining the recombinant boundaries.

### 2.3. Sequencing Assay of the Genes at the Located Locus

Whole-exome sequencing as well as bioinformatical analysis in two affected members (IV-2 and III-1) and one unaffected member (III-2) from Family 1 were fulfilled as described previously [70,71,72,73]. In brief, for each member selected, 5 µg of genomic DNA was sonicated to ~100–900 bp by utilizing the Bioruptor^®^ Plus sonicator (Diagenode, Denville, NJ, USA) to construct a genomic library with the human all exon v5 kit (Agilent Technologies, Santa Clara, CA, USA). The exome library was enriched and subjected to sequencing under the instrument of Illumina HiSeq4000 Genome Analyzer (Illumina, San Diego, CA, USA) as per the manufacturer’s manual. Raw image data were processed, utilizing the software Pipeline (Illumina, San Diego, CA, USA) for base-calling, and the clean sequence reads were aligned with the human genome (GRCh37/hg19) with the software BWA. The software SAMtools was employed to identify sequence variations (single nucleotide variations, deletions, and insertions) within targeted regions. The variants were annotated with the software ANNOVAR, excluding the variants with a minor allele frequency of ≥1% based on the Genome Aggregation Database (gnomAD; http://gnomad.broadinstitute.org, accessed on 8 February 2020) and the Single Nucleotide Polymorphism database (SNP; https://www.ncbi.nlm.nih.gov/SNP, accessed on 8 February 2020). Only the non-synonymous variations (single nucleotide variations, deletions, and insertion) and those generating premature stop codons or alternate splice sites underwent further analysis, encompassing assessment of the pathogenic effect of variations by MutationTaster (https://www.mutationtaster.org, accessed on 9 February 2020) and PolyPhen-2 (http://genetics.bwh.harvard.edu/pph2, accessed on 9 February 2020), Sanger sequencing assay of the coding exons and flanking introns of the genes harboring identified causative variations in the family with CHD, and co-segregation analysis in the CHD family.

### 2.4. Expression Vector Construction and Site-Directed Mutagenesis

The eukaryotic expression vector of human wild-type connexin 45 (Cx45)-IRES-GFP was constructed by inserting human Cx45, which was derived from the pBluescriptII SK(+) vector provided generously by Dr. Eric C. Beyer (at The University of Chicago, Chicago, IL, USA), into pIRES2-EGFP. The human wild-type Cx43-IRES-DsRed plasmid was constructed by inserting Cx43 from the Cx43-IRES-GFP plasmid into the pIRES-DsRed plasmid. The human wild-type Cx45-IRES-DsRed plasmid was constructed by inserting Cx45 from the Cx45-IRES-GFP plasmid into the pIRES2-DsRed2 plasmid. The wild-type Cx45-IRES-GFP plasmids was utilized as a template to produce the R184G-mutant Cx45 plasmid, R184G-IRES-GFP, using a site-directed mutagenesis kit (Stratagene, Santa Clara, CA, USA) and a complementary pair of primers: forward 5’-GCAGTTGCTGGCAGGGACCGTGTTTGAGG-3’ and reverse 5’-CCTCAAACACGGTCCCTGCCAGCAACTGC-3’. The Cx45 and Cx43 sequences in expression plasmids were confirmed by Sanger sequencing assay.

### 2.5. Cell Culture and Transient Transfection

Gap junction-deficient HeLa and N2A cells (from American Type Culture Collection, Manassas, VA, USA) were grown in a Dulbecco’s modified Eagle’s medium (DMEM, Thermo Fisher Scientific, Carlsbad, CA, USA) containing 1% penicillin, 1% streptomycin, 4.5 g/L D-glucose, 110 mg/L sodium pyruvate, 584 mg/L L-glutamine, and 10% fetal bovine serum (Invitrogen, Burlington, ON, Canada), in a thermostat at 37 °C with an atmosphere consisting of 95% air and 5% CO_2_. Cells were plated on 35-mm dishes at 50–60% confluence 24 h prior to cellular transfection. For every transfection experiment, cells were incubated with 1.0 µg of a cDNA plasmid and 2 µL of a transfection reagent (X-tremeGENE HP, Roche Applied Science, Mannheim, Germany) in Opti-MEM (Invitrogen, Burlington, ON, Canada). Approximately 24 h post-transfection, HeLa cells were applied to the dye uptake analysis, and N2A cells were cultured on glass coverslips for about 2.5 h before electrophysiological recording.

### 2.6. Electrophysiological Analysis

The glass coverslips with N2A cells grown on them were placed in a recording chamber and soaked in extracellular solution (ECS) at room temperature (22–25 °C). The recording chamber was placed on a fluorescence microscope (DMIRB, Leica, Wetzlar, Germany) to visualize reporter-positive cell pairs. An isolated pair of cells (with both cells GFP-positive or one GFP-positive and another DsRed-positive) were patched by utilizing two glass micropipettes (with a pipette resistance of 2–4 MΩ), which were filled with intracellular solution (ICS). The compositions of ECS and ICS have been described elsewhere [74,75]. The electrophysiological characteristics of cell pairs expressing wild-type Cx45 or R814G-mutant Cx45 were studied by using the dual voltage-clamp procedure, as previously described [74,75]. To study gap junction coupling and trans-junctional voltage-dependent gating (V_j_-gating) for a selected pair of cells, one was voltage clamped at 0 mV, whereas the other was also voltage clamped with delivery of a series of voltage pulses (7 s duration), from initial ±20 mV to final ±100 mV, with an increment of 20 mV. The trans-junctional current (I_j_) was recorded with Axopatch 200B (Molecular Devices, Sunnyvale, CA, USA) and digitalized at a sampling frequency of 10 kHz through an ADDA converter (Molecular Devices, Sunnyvale, CA, USA). Trans-junctional conductance (G_j_) was calculated with the formula G_j_ = I_j_/V_j_. We routinely perform negative control experiments on cell pairs expressing GFP alone and no GJ coupling in these controls was observed.

### 2.7. Dye Uptake Analysis

The hemichannel function of wild-type Cx45 and R184G-mutant with untagged GFP (Cx45-IRES-GFP and R184G-IRES-GFP) were evaluated by propidium iodide (PI) uptake experiments. In these experiments, the empty IRES-GFP and Cx26-GFP vectors were utilized as the negative and positive controls, respectively. HeLa cells were grown at a lower density, with cells isolated from one another. The cells transfected with plasmids were washed with ECS containing divalent cation, as described previously [74], and were then incubated in a divalent cation-free ECS supplemented with 150 μM of PI at 37 °C for 20 min. After incubation, the cells were washed with ECS containing divalent cation and then observed under a fluorescent microscope (DMIRE2, Leica, Cridersville, OH, USA). The number of cells with or without uptake of PI was counted, and the percentage of cells showing uptake of PI was calculated. Cells in pairs or clusters were completely ruled out to keep off errors caused by gap junction (GJ) channels.

### 2.8. Statistical Assay

Continuous data were tested for normality by using the Kolmogorov–Smirnov test before parametric data analysis was performed. Data with a normal distribution were expressed as means ± standard errors of the means (SEM), and data with a skewed distribution were presented as medians with interquartile ranges. Student’s two-tailed t-test was applied to the comparison of two groups of quantitative data. One-way ANOVA with a Tukey post-hoc test was conducted to compare data with greater than two groups. Fischer’s exact test was performed to make the comparison between two groups of categorical variables. A significant difference is indicated with asterisks on the graphs (*, **, and *** signify *p* < 0.05, *p* < 0.01, and *p* < 0.001, respectively). The results given on the graphs were derived from at least three independent experiments.

## 3. Results

### 3.1. Identification of a Chinese Pedigree Affected with CHD and AVB

**Figure 1:**
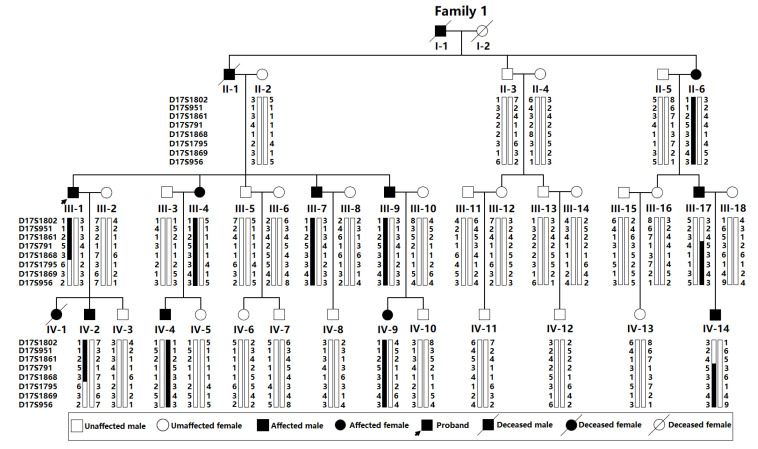
Pedigree and haplotype assay of a large family affected with autosomal dominant congenital heart disease and cardiac arrhythmias. Vertical bars represent the chromosomal segments determined by genotypic analysis with microsatellite markers. Selected microsatellite markers at or near the genetic linkage region on chromosome 17q21.31-q21.33 are exhibited on the left sides of the pedigree, with each family member’s marker-related genotypes given beside the chromosomal bar. A dark vertical bar indicates an affected haplotype, and a white vertical bar indicates a normal haplotype.

A four-generation 40-member Chinese family (Family 1) affected with CHD and AVB, including 36 living family members (16 female members and 20 male members, with ages varying from 12 years to 67 years), was identified, as illustrated in Figure 1.

Clinical investigations of the family members and genetic analyses of the pedigree suggested an apparently autosomal-dominant transmission of CHD and AVB in the family, with complete penetrance. Specifically, all the 13 affected individuals had ASD, and other cardiovascular structural malformations also occurred in some affected individuals from Family 1, including VSD and TOF. All the living patients underwent catheter-based or surgical therapy for CHD. The proband’s daughter (IV-1) died of congestive heart failure at the age of 1 year. Sudden cardiac death occurred in the proband’s father (II-1) and grandfather (I-1) when they were 57 and 62 years old, respectively. Additionally, electrocardiograms demonstrated different degrees of AVB in all individuals with CHD, and sinus bradycardia in three affected individuals. Following-up studies in some individuals indicated progressive cardiac conduction block, and pacemakers were implanted in three affected individuals with third-degree AVB. The basic clinical profiles of the family members suffering CHD and AVB are outlined in Table 1.

### 3.2. A Novel Genetic Locus for CHD and AVB Mapped on Chromosome 17q21.31-q21.33

By genome-wide linkage assay in a family affected with CHD and AVB, we obtained significant evidence suggesting the linkage of CHD and AVB to the marker of D17S1868, with the maximum 2-point LOD score (Zmax) being 3.6124 (at recombination fraction θ = 0.00). To confine the chromosomal region linked to CHD and AVB revealed by the marker of D17S1868, six additional markers (D17S1802, D17S951, D17S1861, D17S791, D17S1869, and D17S956) were used to genotype the family members available, with a Zmax of 4.1399 at marker D17S791 with recombination θ = 0.00, and the haplotypes of Family 1 were constructed using the eight markers, as shown in Figure 1. The crossovers occurring in the two affected individuals of III-17 and III-1 defined the proximal border and distal border of the chromosomal interval, respectively, and the disease-linked haplotype possessed by all the affected family members was determined, which finely mapped the disease locus on chromosome 17q21.31-q21.33 (GRCh38, chr17:44,729,895–49,847,617), a 4.82-cM (~5.12 Mbp) interval bounded by D17S1861 and D17S1795 (as shown in Figure 1). The 2-point LOD scores for the eight microsatellite markers used for the construction of the haplotypes are summarized in Table 2.

### 3.3. Discovery of a Pathogenic Mutation in Cx45

**Figure 2:**
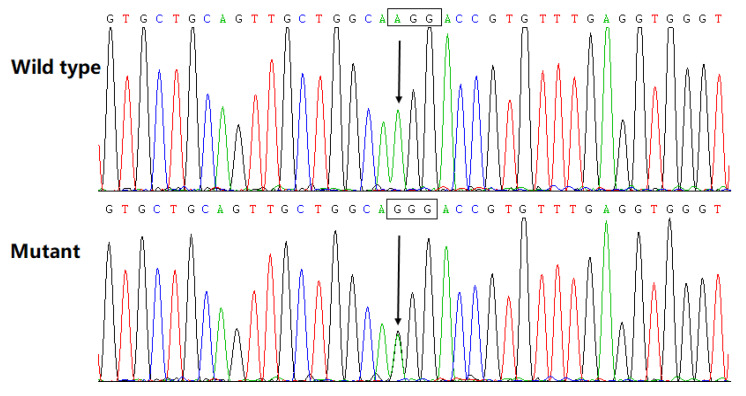
Sequence electropherograms exhibiting the wild-type and heterozygous c.550A>G-mutant *Cx45* alleles. An arrow sign pinpoints the homozygous nucleotides of A/A in an unaffected subject (wild type) or the heterozygous nucleotides of A/G in the affected proband (mutant). A rectangle signal delimits a codon of *Cx45*.

As shown in Table A1, there are 205 genes at the mapped chromosomal locus between the boundaries of markers D17S1861 and D17S1795, including 90 protein-coding genes, 44 pseudogenes, and 71 genes encoding various non-coding RNAs. By whole-exome sequencing and informatics analysis, at the mapped locus, merely the mutation of g.42882636T>A (NC_000017.10), equivalent to g.44805268T>A (NC_000017.11) or NM_005497.4:c.550A>G;p.R184G in the gene of *GJC1*, coding for a protein termed GJ gamma 1 (GJC1) or Cx45, was verified by Sanger sequencing analysis to be in co-segregation with CHD and AVB in the entire family. The missense mutation was neither found in 516 control subjects nor reported in the gnomAD and SNP databases. The sequence chromatograms showing the wild-type and c.550A>G-mutant *Cx45* alleles are displayed in Figure 2.

Additionally, two other rare variants in two other genes in the locus mapped were detected by whole-exome sequencing and informatics analysis, including NM_001145365.3:c.958A>C;p.I320L in *ZNF652*, found in the two affected family members (III-1 and IV-2) and NM_013351.2:c.896>A;p.F299Y in *TBX21*, found in the one unaffected member (III-2). However, neither of the two rare variants co-segregated with the disease in the whole family, suggesting that these two genetic variants are unlikely to cause the disorder.

### 3.4. Impaired Coupling Conductance of Cells Expressing Cx45 R184G

**Figure 3:**
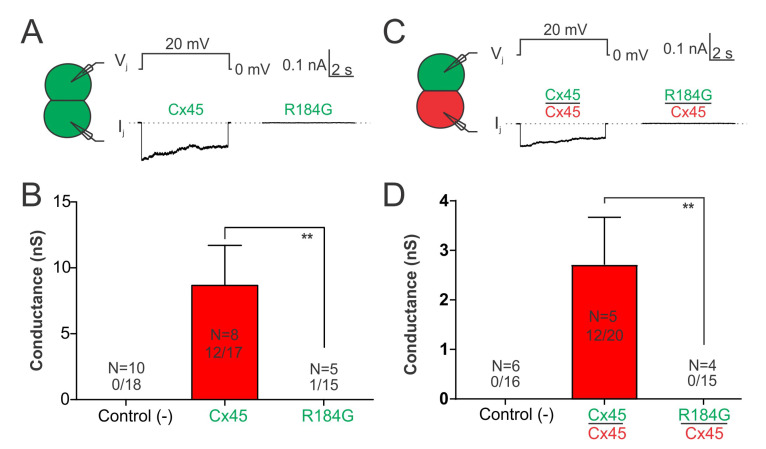
Impaired coupling conductance (G_j_) of homotypic Cx45 R184G GJ channels in N2A cells. (**A**) Representative current recordings on N2A cell pairs that expressed Cx45 R184G showed no trans-junctional current (I_j_) in response to a trans-junctional voltage (V_j_)-pulse; in contrast, cell pairs expressing wild-type Cx45 typically showed I_j_. (**B**) Bar graph to show coupling probability (12/17) and coupling conductance (G_j_) of homotypic Cx45 R184G, together with wildtype Cx45 GJs and the negative control (expressing empty vector, IRES-GFP, alone). The cell pairs that expressed the homomeric Cx45 R184G mutant showed significantly reduced coupling probability and G_j_, in comparison with those expressing wild-type Cx45. (**C**) Representative cell pairs with wild-type Cx45 expressed in one and R184G mutant expressed in the other showed no I_j_ under a V_j_-pulse, in contrast to those with wild-type Cx45 expressed in both cells where I_j_ was observed. (**D**) Bar graph to summarize coupling probability (12/20) and G_j_ of heterotypic R184G/Cx45 GJ channels. The cell pairs that expressed heterotypic R184G/Cx45 GJ showed no coupling, which is significantly different from to the Gj of cells expressing Cx45. N given above or in a bar represents the number of independent transfections.

The functional effect of the R184G mutation in Cx45 on GJ channels was examined in connexin-deficient N2A cells by utilizing the dual patch-clamp technique in whole-cell voltage clamp recording mode. As shown in Figure 3A, the cell pairs expressing wild-type Cx45 typically showed transjunctional current (I_j_) in response to a V_j_-pulse (20 mV), whereas the cell pairs expressing R184G-mutant Cx45 typically showed no I_j_ in response to a V_j_-pulse. As given in Figure 3B, R184G-mutant Cx45 showed no coupling (G_j_ = 0 nS, n = 15, N = 5), compared to that of wild-type Cx45 (G_j_ = 8.7 ± 3.0 nS, n = 17, N = 8, *p* < 0.01). Additionally, the coupling probability observed for R184G (1/15 cell pairs showing coupling) was less than wild-type Cx45 (12/17 cell pairs showing coupling, *p* < 0.001). The functional roles of heterotypic GJ channels assembled by R184G-mutant Cx45 docking to wild-type Cx45 were assessed by utilizing the dual patch clamps in whole-cell status in cell pairs (with R184G-IRES-GFP expressed in one cell and Cx45-IRES-DsRed expressed in the other). Heterotypic R184G/Cx45 cell pairs typically showed no I_j_ under a V_j_-pulse (Figure 3C), which were different from homotypic Cx45/Cx45 cell pairs (with wild-type Cx45-IRES-GFP expressed in one cell and wild-type Cx45-IRES-DsRed expressed in the other). As shown in Figure 3D, the calculated G_j_ of this R184G/Cx45 cell pair was zero for all cell pairs tested (G_j_ = 0 nS, n = 15, N = 4), compared to wild-type Cx45 coupling conductance (G_j_ = 2.7 ± 0.96 nS, n = 20, N = 5, *p* < 0.01). Moreover, the coupling probability for R184G/Cx45 (0/15 cell pairs showing coupling) was less than wild-type Cx45/Cx45 (12/20 cell pairs showing coupling, *p* < 0.01).

### 3.5. Cx45 R184G Impaired GJ Coupling When Paired with Cx43 Expressing Cells

**Figure 4:**
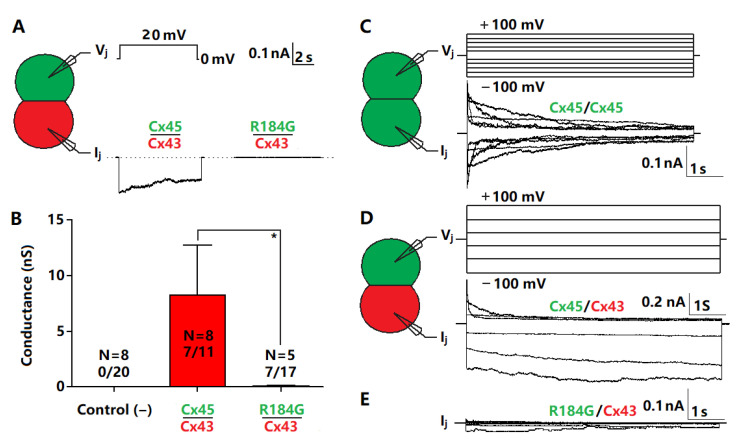
Reduced coupling conductance of heterotypic Cx45 R184G/Cx43 GJ in N2A cells. (**A**) A representative N2A cell pairs with Cx43 expressed in one and Cx45 R184G-mutant expressed in the other showed no trans-junctional current (I_j_) in response to V_j_-pulse, while in a representative cell pair with Cx43 expressed in one and wild-type Cx45 expressed in the other, Ij was recorded. (**B**) Bar graph to show coupling conductance (G_j_) of heterotypic Cx45/Cx43 and Cx45 R184/Cx43 GJs. The cell pairs that expressed R184/Cx43 displayed a significantly decreased G_j_, compared with those expressing Cx45/Cx43 (* *p* < 0.05). N given above or in a bar indicates the number of transfections. (**C**) A representative macroscopic Vj-gating of homotypic Cx45 channels. Macroscopic I_j_ in cell pairs with homotypic Cx45 GJs was illustrated in response to the V_j_-protocol (above), with symmetric deactivations recorded for both Vj polarities. (**D**) Representative macroscopic I_j_s of heterotypic Cx45/Cx43 and (**E**) R184G/Cx43 cell pairs. Superimposed macroscopic I_j_s in cell pairs with heterotypic Cx45/Cx43 or R184G/Cx43 expressed were shown in response to the V_j_-protocol (indicated above). Asymmetric Vj-dependent gating was recorded in both Cx45/Cx43 and R184G/Cx43 GJs, though significantly decreased Ij amplitude was found in R184G/Cx43 cell pairs in contrast to Cx45/Cx43.

The functional effect of heterotypic GJ channels formed by the Cx45 R184G docking to wild-type Cx43 was characterized via utilizing dual whole-cell patch clamp in the cell pairs with R184G-IRES-GFP expressed in one cell and Cx43-IRES-DsRed expressed in the other. Resultantly, Cx45 R184G/Cx43 cell pairs showed very little I_j_ under a V_j_-pulse (Figure 4A), which were different from that of wild-type Cx45/Cx43 cell pairs. As shown in Figure 4B, the calculated G_j_ of the Cx45 R184G/Cx43 cell pair was reduced significantly (0.09 ± 0.04 nS, n = 17, N = 5) in comparison with that of heterotypic Cx45/Cx43 cell pairs (8.3 ± 4.5 nS, n = 11, N = 8, *p* < 0.05). As shown in Figure 4B, the coupling probability for Cx45 R184G/Cx43 (7/17 cell pairs showing coupling) was similar when compared with wild-type Cx45/Cx43 (7/11 cell pairs showing coupling, *p* > 0.05). Additionally, as shown in Figure 4C,D, a series of V_j_-pulses induced Ijs in either homotypic Cx45 GJs or heterotypic Cx45/Cx43 GJs showed expected signature of Vj-gating for these GJs similar to those described earlier (refs). Similar to heterotypic Cx45/Cx43 GJs, the Cx45 R184G/Cx43 GJ also displayed strong asymmetric Vj-gating, though Cx45 R184G/Cx43 cell pairs showed much smaller I_j_s in comparison with the I_j_s of Cx45/Cx43 (Figure 4E).

### 3.6. Dye Uptake of Cells Expressing Cx45 R184G Is Not Different from Wild-Type Cx45 Expressing Cells

**Figure 5:**
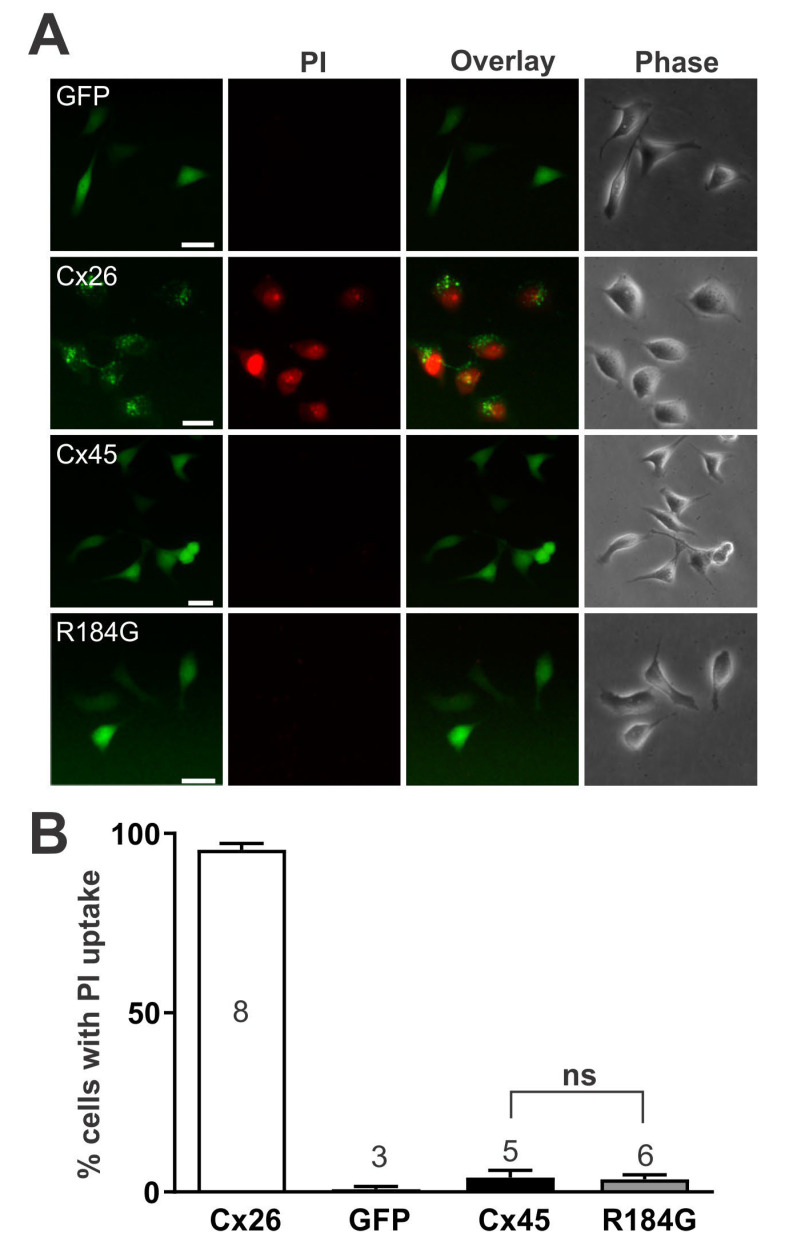
PI uptake in HeLa cells expressing Cx45 R184G was not different from that in wildtype Cx45 expressing cells. (**A**) Representative propidium iodide (PI) uptake fluorescent images of HeLa cells expressing GFP alone (negative control), Cx26-GFP (positive control), wildtype Cx45, and Cx45 R184G. An uptake of little PI was seen for either wild-type Cx45 or Cx45 R184G-expressing cells. The first column exhibits the fluorescence of GFP. The second column exhibits cells with the uptake of PI in red. The third column is an overlay, a combination of red fluorescence and green fluorescence. The fourth column exhibits the phase contrast images of HeLa cells. The white scale bar in the first column images represents 20 µm. (**B**) Bar graphs outlining PI uptake percentage of the HeLa cells that expressed wild-type Cx26-GFP (positive control), GFP (negative control), Cx45, and Cx45 R184G. There was no significant difference (ns) in PI uptake in cells expressing R184G and those expressing wild-type Cx45. N noted above or in a bar is the number of transfections.

Propidium iodide (PI) uptake analysis was conducted to evaluate hemichannel function in HeLa cells expressing Cx45 R184G under a fluorescent microscope. HeLa cells expressing Cx26-GFP showed a prominent PI uptake (showed as red color in Figure 5A). However, little PI uptake was shown for both the wild-type Cx45 and its mutant, R184G (shown in Figure 5A). As shown in Figure 5B, no significant difference in PI uptake was observed between Cx45 R184G (3.5 ± 1.3%, N = 6) and wild-type Cx45 expressing cells (5.7 ± 2.4%, N = 6, *p* > 0.05).

## 4. Discussion

In the current research, a new genetic locus for CHD and AVB was mapped on chromosome 17q21.31-q21.33 by pan-genomic screen with polymorphic markers, linkage analysis, and haplotype assay in a family affected with CHD and arrhythmias. Sequencing analysis of the genes at the locus unveiled a novel mutation in *GJC1* encoding Cx45, c.550A>G (p.R184G), co-segregated with CHD and AVB in the whole family. The heterozygous mutation, which was not found in 1032 control chromosomes, was not reported in such population genetics databases as SNP and gnomAD. Cellular electrophysiological recordings demonstrated that the mutation significantly diminished the coupling conductance in homotypic cell pairs (R184G/R184G, R184G/Cx45) and heterotypic cell pairs (R184G/Cx43), in a dominant-negative mode. These findings suggest that genetically compromised *GJC1*/Cx45 is a novel gene contributing to CHD and AVB.

Connexin, a membrane protein with one intracellular N-terminus, four transmembrane domains, two extracellular loops, andone1 intracellular loop and intracellular C-terminus, is a structural building block of GJ [76]. Six Connexins oligomerized, forming a hemichannel or a connexon, and two connexons from neighboring cells docked together to form a GJ channel, which allows the direct interchange of ions and small molecules (with molecular mass less than 1.2 kDa) between adjacent cells, including metabolites, cAMP, ADP, ATP, nutrients, second messengers, Ca^2+^, and adenosine [76,77,78]. Hence, GJ channels function predominantly to regulate the communication between neighboring cells playing a crucial role in the regulation of embryonic development, physiological homeostasis, and miscellaneous tissue/organ functions [76,77,78,79,80,81]. Notably, the gating of GJ channels is mediated by distinct factors, including trans-junctional voltage (V_j_), intracellular and extracellular Ca^2+^ concentrations, pH changes, reactive oxygen species, phosphorylation, and nitrosylation [78]. In mammals, the connexin gene family consists of 21 members, which are categorized 5 groups, and their proteins are named after their molecular mass, which vary between 23 and 62 kDa (Cx23-Cx62) [76,77]. In the human heart, three isoforms of connexins, including Cx45, Cx40, and Cx43, are highly expressed, with each isotype characterized by a specific spatiotemporal expression pattern and a distinct permeation property [82]. During embryogenesis, Cx45 is the first connexin to be expressed in the heart, while in the hearts of adults, abundant expression of Cx45 is restricted to the auricles as well as the conduction tissue, encompassing the sinoatrial nodes and atrioventricular nodes as well as the His-Purkinje fibers, with less expression of Cx45 in both atria and ventricles [83]. In adults, Cx43 is amply expressed in the ventricles, whereas Cx40 is predominantly expressed in the atria [83]. These connexins may assemble into homomeric or heteromeric hemichannels as well as homotypic or heterotypic GJ channels between adjacent cardiomyocytes, serving to ensure orchestrated intercellular communication and electrical coupling as well as fast propagation of electrical impulse through the heart, therefore having an important role in cardiovascular development and cardiac electrophysiology [83]. In mice, targeted ablation of *Cx45* led to embryonic death, owing to ECD, AVB, and heart failure as well as vascular developmental abnormality [84,85]. Furthermore, myocardium-specific disruption of *Cx45* resulted in the death of murine embryos attributable to ECD and AVB [86]. To avoid the embryonic demise caused by the knockout of *Cx45*, mice were generated with the nullification of *Cx45* induced specifically in the adult murine cardiomyocytes. Although these *Cx45*-null mice were viable, they manifested delayed atrioventricular nodal conduction, with Cx30.2 decreased by about 70% in the mutant hearts [87]. Furthermore, the knockout of both *Cx45* and *Cx30.2* caused a stronger impairment of atrioventricular nodal conductivity in viable mice [87]. In another mouse model with double deficiency of *Cx40*/*Cx45* (*Cx40*^−/−^/*Cx45*^+/−^), various cardiac defects occurred, including VSD, ventricular hypertrophy, AVSD, atrial dilatation, and abnormal myocardial arrangement, which resulted in mortality during embryogenesis and after birth [88]. Meanwhile, either *Cx40*^−/−^ or *Cx45*^+/−^ hearts were structurally normal [88]. Similarly, although the *Cx40*^−/−^ mice manifested prolongation of PQ interval as well as P-wave and QRS-wave duration, and the *Cx45*^+/−^ mice had normal electrocardiographic parameters, the *Cx40*^−/−^/*Cx45*^+/−^ mice showed more significantly prolonged QRS duration and PQ interval in contrast to the *Cx40*^−/−^ mice [88]. In humans, deleterious mutations in *Cx45* were linked to atrial fibrillation and abnormal atrial conduction associated with bone deformations, including sinus bradycardia, atrial standstill, and AVB [70,89]. In this research, a novel malicious mutation in *Cx45* was implicated with CHD and AVB, which expanded the phenotypic spectrum resulting from mutant *Cx45*. These results provide evidence highlighting the essential role of Cx45 in proper cardiovascular development as well as in cardiac electrophysiology.

In mice, knockout of either *Cx40* or *Cx43* has been demonstrated to result in various CHD. In *Cx40*^+/−^ mice, TOF, VSD, bifid atrial appendage, and abnormal aortic arch occurred, while in *Cx40*^−/−^ mice, multiple cardiac malformations including TOF, DORV, and ECD developed [90]. By contrast, in *Cx43*^+/−^ mice, no apparent cardiovascular deformities were observed, while in *Cx43*^−/−^ mice, embryos died at birth, because of outflow tract obstruction and conotruncal cardiac malformations [91]. However, to date, in humans, only mutations in *Cx43* have been reported to give rise to syndromic or non-syndromic CHD, including atrial isomerism, VSD, ASD, and pulmonary stenosis, though mutations in *Cx40*, *Cx43*, and *Cx45* have been implicated with arrhythmias, such as AVB, atrial fibrillation, and sudden infant death syndrome [70,92,93,94]. Provided that the expression spectra and functional features of *Cx45*, *Cx40*, and *Cx43* exist, overlapping in mice and humans, the aforementioned results indicate that mutations in *Cx45* predispose to CHD in humans. Nevertheless, the underlying mechanism of how R184G-mutant Cx45 causes CHD remains to be further elucidated.

The association of genetically compromised *Cx45* with enhanced vulnerability to sinus bradycardias may be at least in part ascribed to the inhibited cardiac conduction tissue, encompassing the sinoatrial nodes and atrioventricular nodes. In the current study, cellular electrophysiological experiments revealed that the R184G mutation diminished trans-junctional electrical coupling in homotypic and heterotypic cell pairs with wild-type Cx43 or Cx45, in a dominant-negative fashion. It was demonstrated that dysfunctional GJ channels in the heart contributed to heterogeneous conduction and decreased conduction velocity [95,96]. Therefore, impaired GJ channel function resulting from genetically defective Cx45 may produce a matrix for bradycardias. The molecular and cellular mechanism of how Cx45 R184G impairs the GJ is not clear. From homology structure models of Cx45, the R184 is in the middle of the third transmembrane domain, likely forming non-covalent interactions with residues in other transmembrane domains. Mutation from a large positively-charged Arg (R) into a small Gly (G) without any sidechain could eliminate the inter-transmembrane domain interactions or increase the flexibility of the α-helix structure; either of these structural changes could impair the normal folding of connexin and the subsequent oligomerization into hemichannels and/or GJ. Misfolded, improper, or incomplete oligomerization of connexin could be trapped intracellularly and unable to reach the plasma membrane to form GJs at the cell–cell interface. One of our previously studied Cx45 mutant (M235L) showed that the M235L was intracellularly distributed and unable to form any morphological GJ plaques at the cell–cell interface [70]. More experiments on the R184G localization would help to see if a similar impairment in localization also happened. Of course, we could not rule out other possible mechanisms of GJ function impairment by this mutation.

Notably, in contrast to our reported clinical phenotypes caused by the Cx45 R184G mutation, no CHD bone malformation, encompassing craniofacial and dentodigital dysplasia in the carriers of the Cx45 R75H mutation, was reported [89]. The remarkable phenotypic discrepancy can be interpreted partially by the following reasons. Firstly, the nature of the two Cx45 mutants is distinct: the R75H mutant dominant-negatively prohibits both electrophysiological and permeable functions of the GJ channels [89], whereas the R184G mutant only suppresses the electrophysiological properties in a dominant-negative pattern, without any detectable change in PI uptake comparing to that of Cx45. Secondly, clinical phenotypes linked to the Cx45 genotypes may have an incomplete penetration. Thirdly, there are diverse genetic backgrounds among affected individuals. Finally, environmental factors may play a role in modifying clinical expressivity, and notably, some of the arrhythmias might have been secondary complications from prior cardiac surgery.

## 5. Conclusions

Conclusively, the present investigation maps a novel genetic locus for CHD and AVB on human chromosome 17q21.31-q21.33 and identifies *Cx45* as a possible new causative gene responsible for CHD and arrhythmias in humans. The findings shed light on the molecular pathogenesis of CHD and arrhythmias and provide evidence highlighting the key role of Cx45 in cardiogenesis and cardiac electrophysiology, implying implications for prognostic risk evaluation and improved management of patients suffering from CHD and arrhythmias.

## Figures and Tables

**Table 1:** Basic clinical information of the affected individuals from Family 1 suffering congenital heart disease and cardiac arrhythmias.

Family Member Information			Cardiac Phenotypes	
Identity (Family 1)	Sex	Years of Age	Cardiac Developmental Deformations	Arrhythmias
I-1 II-1 II-6 III-1 III-4 III-7 III-9 III-17 IV-1 IV-2 IV-4 IV-9 IV-14	M M F M F M M M F M M F M	62 * 57 * 67 47 45 38 35 41 1 * 23 22 12 15	ASD, VSD ASD, VSD ASD ASD ASD ASD ASD ASD ASD, TOF ASD, VSD ASD ASD ASD	AVB (third-degree), SB AVB (third-degree), SB AVB (third-degree), SB AVB (third-degree) AVB (third-degree) AVB (second-degree) AVB (second-degree) AVB (second-degree) AVB (first-degree) AVB (first-degree) AVB (second-degree) AVB (first-degree) AVB (first-degree)

ASD: atrial septal defect; AVB: atrioventricular block; F: female; M: male; SB: sinus bradycardia; TOF: tetralogy of Fallot; VSD: ventricular septal defect. * Age at death.

**Table 2:** Scores of the 2-point logarithm of odds for the eight markers on chromosome 17q21.31-q21.33 at distinct recombination fractions in Family 1 suffering congenital heart disease.

Marker	Scores of the Two-Point Logarithm of Odds for the Markers at *θ* =						
	0.00	0.01	0.05	0.10	0.20	0.30	0.40
D17S1802	(−∞)	1.8523	2.3017	2.2781	1.8844	1.3013	0.6230
D17S951	(−∞)	0.0723	0.6292	0.7465	0.6597	0.4246	0.1479
D17S1861	(−∞)	1.5557	2.0228	2.0228	1.6803	1.1552	0.5438
D17S791	4.2141	4.1399	3.8356	3.4376	2.5823	1.6632	0.7496
D17S1868	3.6124	3.5427	3.2597	2.8978	2.1540	1.3958	0.6421
D17S1795	(−∞)	-0.4400	0.7442	1.0686	1.0783	0.7872	0.3677
D17S1869	(−∞)	1.5513	2.0007	1.9777	1.5901	1.0332	0.4347
D17S956	(−∞)	0.6657	1.1867	1.2570	1.0680	0.7168	0.3063

Θ: recombination fraction.

## Data Availability

All data are included in this paper and Table A1.

## Appendix A

**Table A1:** A total of 205 genes at the mapped chromosomal locus between the boundaries of markers D17S1861 and D17S1795.

DBF4B	LOC107985044 (lncRNA)	ADAM11	GJC1	HIGD1B
**EFTUD2**	RN7SL405P (pseudo)	**CCDC103**	**GFAP**	**FAM187A**
LOC105371793 (lncRNA)	**KIF18B**	MIR6783 (miRNA)	**C1QL1**	LOC112268183 (pseudo)
LOC107987243 (lncRNA)	**DCAKD**	**NMT1**	**PLCD3**	MIR6784 (miRNA)
**ACBD4**	**HEXIM1**	**HEXIM2**	LOC105371795 (lncRNA)	FMNL1-DT (lncRNA)
**FMNL1**	**LOC124904011**	MAP3K14-AS1 (lncRNA)	**SPATA32**	**MAP3K14**
RNA5SP443 (pseudo)	LOC105371796 (lncRNA)	**ARHGAP27**	LOC124904016 (lncRNA)	**PLEKHM1**
MIR4315-1 (miRNA)	LOC124904113 (pseudo)	LOC105369225 (lncRNA)	LRRC37A4P (pseudo)	LOC102724345 (pseudo)
RDM1P1 (pseudo)	DND1P1 (pseudo)	MAPK8IP1P2 (pseudo)	RPS26P8 (pseudo)	LINC02210 (lncRNA)
LINC02210-CRHR1 (lncRNA)	ARF2P (pseudo)	LOC105371802 (lncRNA)	LOC107985028 (lncRNA)	**CRHR1**
MAPT-AS1 (lncRNA)	**SPPL2C**	**MAPT**	MAPT-IT1 (lncRNA)	LOC105371800 (lncRNA)
**STH**	**KANSL1**	LOC107985027 (lncRNA)	KANSL1-AS1 (lncRNA)	MAPK8IP1P1 (pseudo)
**LRRC37A**	DND1P2 (pseudo)	LOC100132570 (pseudo)	LOC124904014 (lncRNA)	**ARL17B**
RN7SL656P (pseudo)	**LRRC37A2**	NSFP1 (pseudo)	**ARL17A**	RDM1P2 (pseudo)
RN7SL199P (pseudo)	**NSF**	LOC107985026 (pseudo)	RPS7P11 (pseudo)	**WNT3**
LOC101929777 (lncRNA)	**WNT9B**	LINC01974 (lncRNA)	LOC112268191 (lncRNA)	RNU6ATAC3P (pseudo)
**GOSR2**	MIR5089 (miRNA)	**RPRML**	LOC101927060 (lncRNA)	LRRC37A17P (pseudo)
RN7SL270P (pseudo)	LOC124904015 (lncRNA)	**CDC27**	LOC112268192 (pseudo)	RPS2P47 (pseudo)
**MYL4**	**ITGB3**	RNU7-186P (pseudo)	LOC107985029 (lncRNA)	EFCAB13-DT (lncRNA)
**EFCAB13**	LOC100421523 (pseudo)	NFE2L3P2 (pseudo)	MRPL45P2 (pseudo)	**NPEPPS**
KPNB1-DT (lncRNA)	**KPNB1**	**TBKBP1**	**TBX21**	**OSBPL7**
**MRPL10**	**LRRC46**	**SCRN2**	**SP6**	SP2-DT (lncRNA)
**SP2**	SP2-AS1 (lncRNA)	**PNPO**	**PRR15L**	**CDK5RAP3**
LOC124904017 (lncRNA)	**COPZ2**	MIR10226 (miRNA)	MIR152 (miRNA)	NFE2L1-DT (lncRNA)
**NFE2L1**	**CBX1**	RNU6-1201P (pseudo)	**SNX11**	**SKAP1**
LOC124904018 (lncRNA)	MIR1203 (miRNA)	RNU6-1152P (pseudo)	LSM3P1 (pseudo)	SKAP1-AS2 (lncRNA)
LOC124904151 (lncRNA)	LOC101927166 (lncRNA)	LOC124904019 (lncRNA)	LOC105371808 (lncRNA)	**HOXB1**
**HOXB2**	HOXB-AS1 (lncRNA)	**HOXB3**	HOXB-AS2 (lncRNA)	**HOXB4**
MIR10A (miRNA)	HOXB-AS3 (lncRNA)	**HOXB5**	**HOXB6**	**HOXB7**
**HOXB8**	**HOXB9**	HOXB-AS4 (lncRNA)	MIR196A1 (miRNA)	LINC02086 (lncRNA)
RPL9P28 (pseudo)	COX6B1P2 (pseudo)	**PRAC2**	**PRAC1**	MIR3185 (miRNA)
**HOXB13**	LOC105371811 (lncRNA)	LOC105371812 (lncRNA)	**TTLL6**	RN7SL125P (pseudo)
LOC105371813 (lncRNA)	**CALCOCO2**	SUMO2P17 (pseudo)	LOC105371814 (lncRNA)	LOC124900395 (lncRNA)
**ATP5MC1**	LOC100419622 (pseudo)	**UBE2Z**	**SNF8**	RNU1-42P (pseudo)
LOC124904116 (lncRNA)	**GIP**	**IGF2BP1**	LOC124904020 (lncRNA)	RNU6-826P (pseudo)
B4GALNT2P1 (pseudo)	**B4GALNT2**	LOC124904021 (lncRNA)	RPS10P25 (pseudo)	TRQ-TTG1-1 (tRNA)
**GNGT2**	**ABI3**	**PHOSPHO1**	LOC124904022 (lncRNA)	FLJ40194 (lncRNA)
**ZNF652**	MIR6129 (miRNA)	LOC124904111 (snRNA)	ZNF652-AS1 (lncRNA)	RPL21P124 (pseudo)
**PHB1**	EIF4EP2 (pseudo)	LINC02075 (lncRNA)	**NGFR**	NGFR-AS1 (lncRNA)
MIR6165 (miRNA)	**NXPH3**	**SPOP**	LOC107984999 (lncRNA)	**SLC35B1**
**FAM117A**	**KAT7**	SRP14P3 (pseudo)	**TAC4**	FLJ45513 (lncRNA)

## References

[1] Zaidi S., Brueckner M. (2017). Genetics and genomics of congenital heart disease. Circ. Res..

[2] Benjamin E.J., Virani S.S., Callaway C.W., Chamberlain A.M., Chang A.R., Cheng S., Chiuve S.E., Cushman M., Delling F.N., Deo R. (2018). American Heart Association Council on Epidemiology and Prevention Statistics Committee and Stroke Statistics Subcommittee. Heart disease and stroke statistics-2018 update: A report from the American Heart Association. Circulation.

[3] Villaseca-Rojas Y., Varela-Melo J., Torres-Castro R., Vasconcello-Castillo L., Mazzucco G., Vilaró J., Blanco I. (2022). Exercise Capacity in Children and Adolescents with Congenital Heart Disease: A Systematic Review and Meta-Analysis. Front. Cardiovasc. Med..

[4] Brudy L., Häcker A.L., Meyer M., Oberhoffer R., Hager A., Ewert P., Müller J. (2022). Adults with Congenital Heart Disease Move Well but Lack Intensity: A Cross-Sectional Study Using Wrist-Worn Physical Activity Trackers. Cardiology.

[5] Brudy L., Meyer M., Garcia-Cuenllas L., Oberhoffer R., Hager A., Ewert P., Müller J. (2021). Objective Physical Activity Assessment in Clinical Congenital Heart Disease Research: A Systematic Review on Study Quality, Methodology, and Outcomes. Cardiology.

[6] Brudy L., Meyer M., Oberhoffer R., Ewert P., Müller J. (2021). Move more–be happier? physical activity and health-related quality of life in children with congenital heart disease. Am. Heart J..

[7] Liu H.C., Chaou C.H., Lo C.W., Chung H.T., Hwang M.S. (2022). Factors Affecting Psychological and Health-Related Quality-of-Life Status in Children and Adolescents with Congenital Heart Diseases. Children.

[8] Sadhwani A., Wypij D., Rofeberg V., Gholipour A., Mittleman M., Rohde J., Velasco-Annis C., Calderon J., Friedman K.G., Tworetzky W. (2022). Fetal Brain Volume Predicts Neurodevelopment in Congenital Heart Disease. Circulation.

[9] Schlatterer S.D., Govindan R.B., Murnick J., Barnett S.D., Lopez C., Donofrio M.T., Mulkey S.B., Limperopoulos C., du Plessis A.J. (2022). In infants with congenital heart disease autonomic dysfunction is associated with pre-operative brain injury. Pediatr. Res..

[10] Parekh S.A., Cox S.M., Barkovich A.J., Chau V., Steurer M.A., Xu D., Miller S.P., McQuillen P.S., Peyvandi S. (2022). The Effect of Size and Asymmetry at Birth on Brain Injury and Neurodevelopmental Outcomes in Congenital Heart Disease. Pediatr. Cardiol..

[11] Yeh H.R., Kim E.H., Yu J.J., Yun T.J., Ko T.S., Yum M.S. (2022). Arterial ischemic stroke in children with congenital heart diseases. Pediatr. Int..

[12] Giang K.W., Fedchenko M., Dellborg M., Eriksson P., Mandalenakis Z. (2021). Burden of Ischemic Stroke in Patients with Congenital Heart Disease: A Nationwide, Case-Control Study. J. Am. Heart Assoc..

[13] Rosenzweig E.B., Krishnan U. (2021). Congenital Heart Disease-Associated Pulmonary Hypertension. Clin. Chest Med..

[14] Jansen K., Constantine A., Condliffe R., Tulloh R., Clift P., Moledina S., Wort S.J., Dimopoulos K. (2021). Pulmonary arterial hypertension in adults with congenital heart disease: Markers of disease severity, management of advanced heart failure and transplantation. Expert Rev. Cardiovasc. Ther..

[15] Abassi H., Gavotto A., Picot M.C., Bertet H., Matecki S., Guillaumont S., Moniotte S., Auquier P., Moreau J., Amedro P. (2019). Impaired pulmonary function and its association with clinical outcomes, exercise capacity and quality of life in children with congenital heart disease. Int. J. Cardiol..

[16] Alonso-Gonzalez R., Borgia F., Diller G.P., Inuzuka R., Kempny A., Martinez-Naharro A., Tutarel O., Marino P., Wustmann K., Charalambides M. (2013). Abnormal lung function in adults with congenital heart disease: Prevalence, relation to cardiac anatomy, and association with survival clinical perspective. Circulation.

[17] Dimopoulos K., Diller G.P., Koltsida E., Pijuan-Domenech A., Papadopoulou S.A., Babu-Narayan S.V., Salukhe T.V., Piepoli M.F., Poole-Wilson P.A., Best N. (2008). Prevalence, predictors, and prognostic value of renal dysfunction in adults with congenital heart disease. Circulation.

[18] Gillesén M., Fedchenko M., Giang K.W., Dimopoulos K., Eriksson P., Dellborg M., Mandalenakis Z. (2022). Chronic kidney disease in patients with congenital heart disease: A nationwide, register-based cohort study. Eur. Heart J. Open.

[19] Kourelis G., Kanakis M., Samanidis G., Tzannis K., Bobos D., Kousi T., Apostolopoulou S., Kakava F., Kyriakoulis K., Bounta S. (2022). Acute Kidney Injury Predictors and Outcomes after Cardiac Surgery in Children with Congenital Heart Disease: An Observational Cohort Study. Diagnostics.

[20] Chen H., Ke Q., Weng G., Bao J., Huang J., Yan L., Zheng F. (2021). Risk factors of postoperative acute kidney injury in patients with complex congenital heart disease and significance of early detection of serum transcription factor Nkx2.5. Am. J. Transl. Res..

[21] Cahill T.J., Jewell P.D., Denne L., Franklin R.C., Frigiola A., Orchard E., Prendergast B.D. (2019). Contemporary epidemiology of infective endocarditis in patients with congenital heart disease: A UK prospective study. Am. Heart J..

[22] Tutarel O., Alonso-Gonzalez R., Montanaro C., Schiff R., Uribarri A., Kempny A., Grübler M.R., Uebing A., Swan L., Diller G.P. (2018). Infective endocarditis in adults with congenital heart disease remains a lethal disease. Heart.

[23] Maser M., Freisinger E., Bronstein L., Köppe J., Orwat S., Kaleschke G., Baumgartner H., Diller G.P., Lammers A. (2021). Frequency, Mortality, and Predictors of Adverse Outcomes for Endocarditis in Patients with Congenital Heart Disease: Results of a Nationwide Analysis including 2512 Endocarditis Cases. J. Clin. Med..

[24] Snygg-Martin U., Giang K.W., Dellborg M., Robertson J., Mandalenakis Z. (2021). Cumulative Incidence of Infective Endocarditis in Patients with Congenital Heart Disease: A Nationwide, Case-Control Study Over Nine Decades. Clin. Infect. Dis..

[25] Well A., Mizrahi M., Johnson G., Patt H., Fraser C.D., Mery C.M., Beckerman Z. (2022). Aortic dissection and ruptures in adult congenital heart disease in Texas from 2009 to 2019. Eur. J. Cardiothorac. Surg..

[26] Arnaert S., De Meester P., Troost E., Droogne W., Van Aelst L., Van Cleemput J., Voros G., Gewillig M., Cools B., Moons P. (2021). Heart failure related to adult congenital heart disease: Prevalence, outcome and risk factors. ESC Heart Fail..

[27] Brida M., Lovrić D., Griselli M., Riesgo Gil F., Gatzoulis M.A. (2022). Heart failure in adults with congenital heart disease. Int. J. Cardiol..

[28] Kartas A., Papazoglou A.S., Kosmidis D., Moysidis D.V., Baroutidou A., Doundoulakis I., Despotopoulos S., Vrana E., Koutsakis A., Rampidis G.P. (2022). The Adult Congenital Heart Disease Anatomic and Physiological Classification: Associations with Clinical Outcomes in Patients with Atrial Arrhythmias. Diagnostics.

[29] Casteigt B., Samuel M., Laplante L., Shohoudi A., Apers S., Kovacs A.H., Luyckx K., Thomet C., Budts W., Enomoto J. (2021). Atrial arrhythmias and patient-reported outcomes in adults with congenital heart disease: An international study. Heart Rhythm.

[30] Wasmer K., Eckardt L., Baumgartner H., Köbe J. (2021). Therapy of supraventricular and ventricular arrhythmias in adults with congenital heart disease-narrative review. Cardiovasc. Diagn. Ther..

[31] Khairy P., Silka M.J., Moore J.P., DiNardo J.A., Vehmeijer J.T., Sheppard M.N., van de Bruaene A., Chaix M.A., Brida M., Moore B.M. (2022). Sudden cardiac death in congenital heart disease. Eur. Heart J..

[32] Van Bulck L., Goossens E., Morin L., Luyckx K., Ombelet F., Willems R., Budts W., De Groote K., De Backer J., Annemans L. (2022). Last year of life of adults with congenital heart diseases: Causes of death and patterns of care. Eur. Heart J..

[33] Constantine A., Costola G., Bianchi P., Chessa M., Giamberti A., Kempny A., Rafiq I., Babu-Narayan S.V., Gatzoulis M.A., Hoschtitzky A. (2021). Enhanced Assessment of Perioperative Mortality Risk in Adults with Congenital Heart Disease. J. Am. Coll. Cardiol..

[34] Vehmeijer J.T., Koyak Z., Leerink J.M., Zwinderman A.H., Harris L., Peinado R., Oechslin E.N., Robbers-Visser D., Groenink M., Boekholdt S.M. (2021). Identification of patients at risk of sudden cardiac death in congenital heart disease: The PRospEctiVE study on implaNTable cardIOverter defibrillator therapy and suddeN cardiac death in Adults with Congenital Heart Disease (PREVENTION-ACHD). Heart Rhythm.

[35] Triedman J.K., Newburger J.W. (2016). Trends in congenital heart disease: The next decade. Circulation.

[36] McCracken C., Spector L.G., Menk J.S., Knight J.H., Vinocur J.M., Thomas A.S., Oster M.E., St Louis J.D., Moller J.H., Kochilas L. (2018). Mortality following pediatric congenital heart surgery: An analysis of the causes of death derived from the National Death Index. J. Am. Heart Assoc..

[37] Fuchs S.R., Smith A.H., Van Driest S.L., Crum K.F., Edwards T.L., Kannankeril P.J. (2019). Incidence and effect of early postoperative ventricular arrhythmias after congenital heart surgery. Heart Rhythm.

[38] Hill M.C., Kadow Z.A., Long H., Morikawa Y., Martin T.J., Birks E.J., Campbell K.S., Nerbonne J., Lavine K., Wadhwa L. (2022). Integrated multi-omic characterization of congenital heart disease. Nature.

[39] Maskell L.J., Qamar K., Babakr A.A., Hawkins T.A., Heads R.J., Budhram-Mahadeo V.S. (2017). Essential but partially redundant roles for POU4F1/Brn-3a and POU4F2/Brn-3b transcription factors in the developing heart. Cell Death Dis..

[40] Nie X., Liu X., Wang C., Wu Z., Sun Z., Su J., Yan R., Peng Y., Yang Y., Wang C. (2022). Assessment of evidence on reported non-genetic risk factors of congenital heart defects: The updated umbrella review. BMC Pregnancy Childbirth.

[41] Han X., Wang B., Jin D., Liu K., Wang H., Chen L., Zu Y. (2021). Precise Dose of Folic Acid Supplementation Is Essential for Embryonic Heart Development in Zebrafish. Biology.

[42] Lim T.B., Foo S.Y.R., Chen C.K. (2021). The Role of Epigenetics in Congenital Heart Disease. Genes.

[43] García-Flores E., Rodríguez-Pérez J.M., Borgonio-Cuadra V.M., Vargas-Alarcón G., Calderón-Colmenero J., Sandoval J.P., García-Montes J.A., Espinoza-Gutiérrez V.M., Reyes-García J.G., Cazarín-Santos B.G. (2022). DNA Methylation Levels of the *TBX5* Gene Promoter Are Associated with Congenital Septal Defects in Mexican Paediatric Patients. Biology.

[44] Martin L.J., Benson D.W. (2021). Focused Strategies for Defining the Genetic Architecture of Congenital Heart Defects. Genes.

[45] Diab N.S., Barish S., Dong W., Zhao S., Allington G., Yu X., Kahle K.T., Brueckner M., Jin S.C. (2021). Molecular Genetics and Complex Inheritance of Congenital Heart Disease. Genes.

[46] Choudhury T.Z., Garg V. (2022). Molecular genetic mechanisms of congenital heart disease. Curr. Opin. Genet. Dev..

[47] Pierpont M.E., Brueckner M., Chung W.K., Garg V., Lacro R.V., McGuire A.L., Mital S., Priest J.R., Pu W.T., Roberts A. (2018). Genetic basis for congenital heart disease: Revisited: A scientific statement from the American Heart Association. Circulation.

[48] Wang C., Lv H., Ling X., Li H., Diao F., Dai J., Du J., Chen T., Xi Q., Zhao Y. (2021). Association of assisted reproductive technology, germline de novo mutations and congenital heart defects in a prospective birth cohort study. Cell Res..

[49] Lahrouchi N., Postma A.V., Salazar C.M., De Laughter D.M., Tjong F., Piherová L., Bowling F.Z., Zimmerman D., Lodder E.M., Ta-Shma A. (2021). Biallelic loss-of-function variants in PLD1 cause congenital right-sided cardiac valve defects and neonatal cardiomyopathy. J. Clin. Invest..

[50] Ekure E.N., Adeyemo A., Liu H., Sokunbi O., Kalu N., Martinez A.F., Owosela B., Tekendo-Ngongang C., Addissie Y.A., Olusegun-Joseph A. (2021). Exome Sequencing and Congenital Heart Disease in Sub-Saharan Africa. Circ. Genom. Precis. Med..

[51] Audain E., Wilsdon A., Breckpot J., Izarzugaza J.M.G., Fitzgerald T.W., Kahlert A.K., Sifrim A., Wünnemann F., Perez-Riverol Y., Abdul-Khaliq H. (2021). Integrative analysis of genomic variants reveals new associations of candidate haploinsufficient genes with congenital heart disease. PLoS Genet..

[52] van Walree E.S., Dombrowsky G., Jansen I.E., Mirkov M.U., Zwart R., Ilgun A., Guo D., Clur S.B., Amin A.S., Savage J.E. (2021). Germline variants in HEY2 functional domains lead to congenital heart defects and thoracic aortic aneurysms. Genet. Med..

[53] Fu F., Li R., Lei T.Y., Wang D., Yang X., Han J., Pan M., Zhen L., Li J., Li F.T. (2021). Compound heterozygous mutation of the ASXL3 gene causes autosomal recessive congenital heart disease. Hum. Genet..

[54] Massadeh S., Albeladi M., Albesher N., Alhabshan F., Kampe K.D., Chaikhouni F., Kabbani M.S., Beetz C., Alaamery M. (2021). Novel Autosomal Recessive Splice-Altering Variant in *PRKD1* Is Associated with Congenital Heart Disease. Genes.

[55] Basel-Salmon L., Ruhrman-Shahar N., Barel O., Hagari O., Marek-Yagel D., Azulai N., Bazak L., Svirsky R., Reznik-Wolf H., Lidzbarsky G.A. (2021). Biallelic variants in ETV2 in a family with congenital heart defects, vertebral abnormalities and preaxial polydactyly. Eur. J. Med. Genet..

[56] Zhao L., Jiang W.F., Yang C.X., Qiao Q., Xu Y.J., Shi H.Y., Qiu X.B., Wu S.H., Yang Y.Q. (2021). SOX17 loss-of-function variation underlying familial congenital heart disease. Eur. J. Med. Genet..

[57] Hao L., Ma J., Wu F., Ma X., Qian M., Sheng W., Yan T., Tang N., Jiang X., Zhang B. (2022). WDR62 variants contribute to congenital heart disease by inhibiting cardiomyocyte proliferation. Clin. Transl. Med..

[58] Zhou Y., Bai K., Wang Y., Meng Z., Zhou S., Jiang S., Wang H., Wang J., Yang M., Wang Q. (2022). Identification of Rare Variants in Right Ventricular Outflow Tract Obstruction Congenital Heart Disease by Whole-Exome Sequencing. Front. Cardiovasc. Med..

[59] Peng R., Li B., Chen S., Shi Z., Yu L., Gao Y., Yang X., Lu L., Wang H. (2022). Deleterious Rare Mutations of *GLI1* Dysregulate Sonic Hedgehog Signaling in Human Congenital Heart Disease. Front. Cardiovasc. Med..

[60] Delea M., Massara L.S., Espeche L.D., Bidondo M.P., Barbero P., Oliveri J., Brun P., Fabro M., Galain M., Fernández C.S. (2022). Genetic Analysis Algorithm for the Study of Patients with Multiple Congenital Anomalies and Isolated Congenital Heart Disease. Genes.

[61] Meerschaut I., Steyaert W., Bové T., François K., Martens T., De Groote K., De Wilde H., Muiño Mosquera L., Panzer J., Vandekerckhove K. (2022). Exploring the Mutational Landscape of Isolated Congenital Heart Defects: An Exome Sequencing Study Using Cardiac DNA. Genes.

[62] Okashah S., Vasudeva D., El Jerbi A., Khodjet-El-Khil H., Al-Shafai M., Syed N., Kambouris M., Udassi S., Saraiva L.R., Al-Saloos H. (2022). Investigation of Genetic Causes in Patients with Congenital Heart Disease in Qatar: Findings from the Sidra Cardiac Registry. Genes.

[63] Shi H.Y., Xie M.S., Yang C.X., Huang R.T., Xue S., Liu X.Y., Xu Y.J., Yang Y.Q. (2022). Identification of *SOX18* as a New Gene Predisposing to Congenital Heart Disease. Diagnostics.

[64] Huang R.T., Guo Y.H., Yang C.X., Gu J.N., Qiu X.B., Shi H.Y., Xu Y.J., Xue S., Yang Y.Q. (2022). SOX7 loss-of-function variation as a cause of familial congenital heart disease. Am. J. Transl. Res..

[65] Xu Z.Q., Chen W.C., Li Y.J., Suo M.J., Tian G.X., Sheng W., Huang G.Y. (2022). PTPN11 Gene Mutations and Its Association with the Risk of Congenital Heart Disease. Dis. Markers.

[66] Gong L., Wang C., Xie H., Gao J., Li T., Qi S., Wang B., Wang J. (2022). Identification of a novel heterozygous SOX9 variant in a Chinese family with congenital heart disease. Mol. Genet. Genomic. Med..

[67] Wang Z., Qiao X.H., Xu Y.J., Liu X.Y., Huang R.T., Xue S., Qiu H.Y., Yang Y.Q. (2022). SMAD1 Loss-of-Function Variant Responsible for Congenital Heart Disease. Biomed Res. Int..

[68] Abhinav P., Zhang G.F., Zhao C.M., Xu Y.J., Wang J., Yang Y.Q. (2022). A novel *KLF13* mutation underlying congenital patent ductus arteriosus and ventricular septal defect, as well as bicuspid aortic valve. Exp. Ther. Med..

[69] Ke Z.P., Zhang G.F., Guo Y.H., Sun Y.M., Wang J., Li N., Qiu X.B., Xu Y.J., Yang Y.Q. (2022). A novel PRRX1 loss-of-function variation contributing to familial atrial fibrillation and congenital patent ductus arteriosus. Genet. Mol. Biol..

[70] Li R.G., Xu Y.J., Ye W.G., Li Y.J., Chen H., Qiu X.B., Yang Y.Q., Bai D. (2021). Connexin45 (GJC1) loss-of-function mutation contributes to familial atrial fibrillation and conduction disease. Heart Rhythm.

[71] Guo X.J., Qiu X.B., Wang J., Guo Y.H., Yang C.X., Li L., Gao R.F., Ke Z.P., Di R.M., Sun Y.M. (2021). PRRX1 Loss-of-Function Mutations Underlying Familial Atrial Fibrillation. J. Am. Heart Assoc..

[72] Guo Y.H., Wang J., Guo X.J., Gao R.F., Yang C.X., Li L., Sun Y.M., Qiu X.B., Xu Y.J., Yang Y.Q. (2022). KLF13 Loss-of-Function Mutations Underlying Familial Dilated Cardiomyopathy. J. Am. Heart Assoc..

[73] Aboagye E.T., Adadey S.M., Esoh K., Jonas M., de Kock C., Amenga-Etego L., Awandare G.A., Wonkam A. (2022). Age Estimate of *GJB2*-p.(Arg143Trp) Founder Variant in Hearing Impairment in Ghana, Suggests Multiple Independent Origins across Populations. Biology.

[74] Ye W.G., Yue B., Aoyama H., Kim N.K., Cameron J.A., Chen H., Bai D. (2017). Junctional delay, frequency, and direction-dependent uncoupling of human heterotypic Cx45/Cx43 gap junction channels. J. Mol. Cell. Cardiol..

[75] Santos-Miranda A., Chen H., Chen R.C., Odoko-Ishimoto M., Aoyama H., Bai D. (2020). The amino terminal domain plays an important role in transjunctional voltage-dependent gating kinetics of Cx45 gap junctions. J. Mol. Cell. Cardiol..

[76] Garcia-Vega L., O’Shaughnessy E.M., Albuloushi A., Martin P.E. (2021). Connexins and the Epithelial Tissue Barrier: A Focus on Connexin 26. Biology.

[77] Zhu Y. (2022). Gap Junction-Dependent and -Independent Functions of Connexin43 in Biology. Biology.

[78] Zhang Y., Khan S., Liu Y., Siddique R., Zhang R., Yong V.W., Xue M. (2021). Gap Junctions and Hemichannels Composed of Connexins and Pannexins Mediate the Secondary Brain Injury Following Intracerebral Hemorrhage. Biology.

[79] Torrisi F., Alberghina C., Lo Furno D., Zappalà A., Valable S., Li Volti G., Tibullo D., Vicario N., Parenti R. (2021). Connexin 43 and Sonic Hedgehog Pathway Interplay in Glioblastoma Cell Proliferation and Migration. Biology.

[80] Li X. (2021). Seeing Is Believing: Gap Junctions in Motion. Biology.

[81] Peng B., Xu C., Wang S., Zhang Y., Li W. (2022). The Role of Connexin Hemichannels in Inflammatory Diseases. Biology.

[82] Guo Y.H., Yang Y.Q. (2022). Atrial Fibrillation: Focus on Myocardial Connexins and Gap Junctions. Biology.

[83] Severs N.J., Bruce A.F., Dupont E., Rothery S. (2008). Remodelling of gap junctions and connexin expression in diseased myocardium. Cardiovasc. Res..

[84] Kumai M., Nishii K., Nakamura K., Takeda N., Suzuki M., Shibata Y. (2000). Loss of connexin45 causes a cushion defect in early cardiogenesis. Development.

[85] Krüger O., Plum A., Kim J.S., Winterhager E., Maxeiner S., Hallas G., Kirchhoff S., Traub O., Lamers W.H., Willecke K. (2000). Defective vascular development in connexin 45-deficient mice. Development.

[86] Nishii K., Seki A., Kumai M., Morimoto S., Miwa T., Hagiwara N., Shibata Y., Kobayashi Y. (2016). Connexin45 contributes to global cardiovascular development by establishing myocardial impulse propagation. Mech. Dev..

[87] Frank M., Wirth A., Andrié R.P., Kreuzberg M.M., Dobrowolski R., Seifert G., Offermanns S., Nickenig G., Willecke K., Schrickel J.W. (2012). Connexin45 provides optimal atrioventricular nodal conduction in the adult mouse heart. Circ. Res..

[88] Krüger O., Maxeiner S., Kim J.S., van Rijen H.V., de Bakker J.M., Eckardt D., Tiemann K., Lewalter T., Ghanem A., Lüderitz B. (2006). Cardiac morphogenetic defects and conduction abnormalities in mice homozygously deficient for connexin40 and heterozygously deficient for connexin45. J. Mol. Cell. Cardiol..

[89] Seki A., Ishikawa T., Daumy X., Mishima H., Barc J., Sasaki R., Nishii K., Saito K., Urano M., Ohno S. (2017). Progressive atrial conduction defects associated with bone malformation caused by a connexin-45 mutation. J. Am. Coll. Cardiol..

[90] Gu H., Smith F.C., Taffet S.M., Delmar M. (2003). High incidence of cardiac malformations in connexin40-deficient mice. Circ. Res..

[91] Reaume A.G., de Sousa P.A., Kulkarni S., Langille B.L., Zhu D., Davies T.C., Juneja S.C., Kidder G.M., Rossant J. (1995). Cardiac malformation in neonatal mice lacking connexin43. Science.

[92] Britz-Cunningham S.H., Shah M.M., Zuppan C.W., Fletcher W.H. (1995). Mutations of the Connexin43 gap-junction gene in patients with heart malformations and defects of laterality. N. Engl. J. Med..

[93] Molica F., Meens M.J., Morel S., Kwak B.R. (2014). Mutations in cardiovascular connexin genes. Biol. Cell.

[94] Lübkemeier I., Bosen F., Kim J.S., Sasse P., Malan D., Fleischmann B.K., Willecke K. (2015). Human Connexin43E42K mutation from a sudden infant death victim leads to impaired ventricular activation and neonatal death in mice. Circ. Cardiovasc. Genet..

[95] Jansen J.A., van Veen T.A., de Bakker J.M., van Rijen H.V. (2010). Cardiac connexins and impulse propagation. J. Mol. Cell. Cardiol..

[96] Donahue J.K. (2012). Connexin gene transfer preserves conduction velocity and prevents atrial fibrillation. Circulation.

